# Effective radiotherapeutic treatment intensification in patients with pancreatic cancer: higher doses alone, higher RBE or both?

**DOI:** 10.1186/s13014-017-0945-2

**Published:** 2017-12-27

**Authors:** Constantin Dreher, Daniel Habermehl, Oliver Jäkel, Stephanie E. Combs

**Affiliations:** 1 Department of Radiation Oncology, University Hospital Rechts der Isar, Technical University Munich (TUM), Ismaninger Str. 22 Munich, Germany; 2 Department of Radiation Sciences (DRS), Institute of Innovative Radiotherapy (iRT), Helmholtz Zentrum München, Oberschleißheim, Germany; 3 Deutsches Konsortium für Translationale Krebsforschung (DKTK), Partner Site München, München, Germany; 4 Department of Medical Physics in Radiation Oncology, German Cancer Research Center, INF, 280 Heidelberg, Germany; 5 Heidelberg Ion Beam Therapy Center (HIT), INF 450, 69120 Heidelberg, Germany

**Keywords:** Pancreatic cancer, RBE, Ion beam therapy, Protons, Carbon ions, Particle radiotherapy

## Abstract

Pancreatic cancer, especially in case of locally advanced stage has a poor prognosis. Radiotherapy in general can lead to tumor volume reduction, but further improvements, such as ion beam therapy have to be promoted in order to enable dose escalation, which in turn results in better local control rates and downsizing of the tumor itself. Ion beam therapy with its highly promising physical properties is also accompanied by distinct inter- and intrafractional challenges in case of robustness. First clinical results are promising, but further research in motion mitigation and biological treatment planning is necessary, in order to determine the best clinical rationales and conditions of ion beam therapy of pancreatic cancer. This review summarizes the current knowledge and studies on ion beam therapy of pancreatic cancer.

## Exploring the rationale for particle radiotherapy in pancreatic cancer

Pancreatic cancer is one of the most devastating tumors in oncology. With median survival times of about 2 years – after complete resection – and survival times of a few months in the metastasized situation, novel treatment concepts are urgently required. In the setting of non-metastasized, locally advanced pancreatic cancer (LAPC), intensification of local treatment can lead to size reduction enabling complete surgical resection – which correlated significantly with outcome [1, 2]. The role of conventional normofractionated radiotherapy with simultaneous chemotherapy has recently lost importance because of the very effective FOLFIRINOX regime [3]. Nevertheless, sequential radiotherapy and combined chemoradiation is frequently used in case of persistent non-resectability after FOLFIRINOX and often contributes to a secondary resectability with negative resection margins [4, 5]. An evolving new photon-based concept in pancreatic cancer treatment beside intensity-modulated radiotherapy (IMRT) and volumetric-modulated arc therapy (VMAT) is stereotactic body radiotherapy (SBRT). SBRT uses higher single doses and first studies have proven the efficacy of this concept, which could even lead to better treatment results than conventional chemoradiation regimes [5–8]. Due to the known dose-response-relationship in pancreatic cancer, increased dose deposition is necessary, which is possible with modern radiation techniques [9]. In this context dose painting is also of utmost importance, enabling an increase or decrease of the delivered dose, e.g. in regions of high PET signals and vessel involvement or adjusted organs at risks (OAR) [10–12].

Nevertheless, photon-based radiotherapy cannot exceed the physical properties of particle radiotherapy. Both proton and carbon ion radiotherapy are characterized by physical properties, that lead to a dose decline within the entry channel and a high local dose deposition in the Spread Out Bragg Peak (SOBP) region, that is to say high dose deposition in the target volume while at the same time little dose exposure to the surrounding normal tissues [13]. Furthermore, high-LET (linear energy transfer) radiotherapy, such as carbon ion radiotherapy is characterized by high energy deposition in the trajectory, resulting in clustered double-strand breaks in the cells’ DNA (Deoxyribonucleic acid) and the generation of bulky lesions [14–16]. So, carbon and oxygen ion beams have a higher relative biological effectiveness (RBE) than photon and proton beams, thus leading to an enhanced reduction in clonogenic survival of pancreatic and also of hepatic cell lines [14, 15, 17–19]. El Shafie et al. at HIT could show, that on the one hand clonogenic survival is directly dependent on the dose, and on the other hand pancreatic cancer is characterized by high radioresistance in case of photon, but not heavier particle beams [14]. So, high-LET beams can overcome the tissue’s radioresistance for photons - this can be partly explained by the hypoxic metabolism of pancreatic cancer. High-LET is characterized by a reduced oxygen enhancement ratio, thus leading to high efficacy against hypoxic tumors [20, 21]. With regard to these results, particle beams seem to be promising.

With highly radiosensitive normal tissues surrounding hepatobiliary and pancreatic malignancies, ion beams are thought to be of special interest in this setting because they offer the possibility to significantly reduce dose to the small intestine [22, 23]. However, gastrointestinal toxicity is still a major issue in high dose regions and caution must still be given when introducing combined treatment protocols with chemotherapy [24]. First clinical results, although mostly of retrospective nature, are promising and the aim of this article is to summarize the current knowledge and possible clinical rationales for ion beam therapy of pancreatic cancer.

## Treatment planning

### Treatment planning system

The ion beam facilities around the world are using different treatment planning systems (TPS) with different dose calculation models. For example, at the heavy-ion medical accelerator in Chiba (HIMAC) a treatment planning system based on the first calculations of Sihver et al. was established [25, 26]. Ion beam radiotherapy of pancreatic cancer is based on passive scattering so far and assumes an average RBE of 3.0 at mid-SOBP for all tissues [27]. Nevertheless, there are first planning studies on the basis of scanning ion beam radiotherapy of pancreatic cancer at HIMAC [27–30]. At HIT treatment planning is performed using the raster-scanning technique [31]. The TPS called “*Syngo RT Planning”* (Siemens, Erlangen, Germany) uses the effective dose calculation model (Local Effect Model, LEM) as described by Krämer & Scholz [32]. This dose calculation model has already been established at the “Gesellschaft für Schwerionenforschung” (GSI) and has been integrated in the TPS TrIP [33]. In case of proton beams a fixed RBE value of 1.1 is assumed, even if there is still discussion about whether the value is adequate [34]. The RBE of carbon ion beams depends on different factors, including the $$ \raisebox{1ex}{$\alpha $}\!\left/ \!\raisebox{-1ex}{$\beta $}\right. $$-value, which is the main input parameter for LEM and its dose calculations [35].

### Target delineation

The gross tumor volume (GTV) is delineated as the macroscopic tumor in the treatment planning computed tomography (CT) scan. By including elective microscopic expansion the clinical tumor volume (CTV) is created. The planning target volume (PTV) is including the elective local lymph node area and dose delivery uncertainties. Dose prescription in photon radiotherapy is often defined as 50.4 Gy for the PTV, followed by a “boost” irradiation of an expanded GTV (by 2–4 mm) [2, 36]. In case of ion beam therapy, no homogenous target description has been established, although, the target delineation in high-LET radiotherapy is of utmost importance. With ion beam therapy being able to irradiate with very sharp dose gradients, uncertainty in dose delivery is even more important than in case of photon radiotherapy, which is why ion beam radiotherapy has to include the concept of PTV, analogically to Japanese reports [27, 37, 38].

### Beam setups

**Fig. 1:**
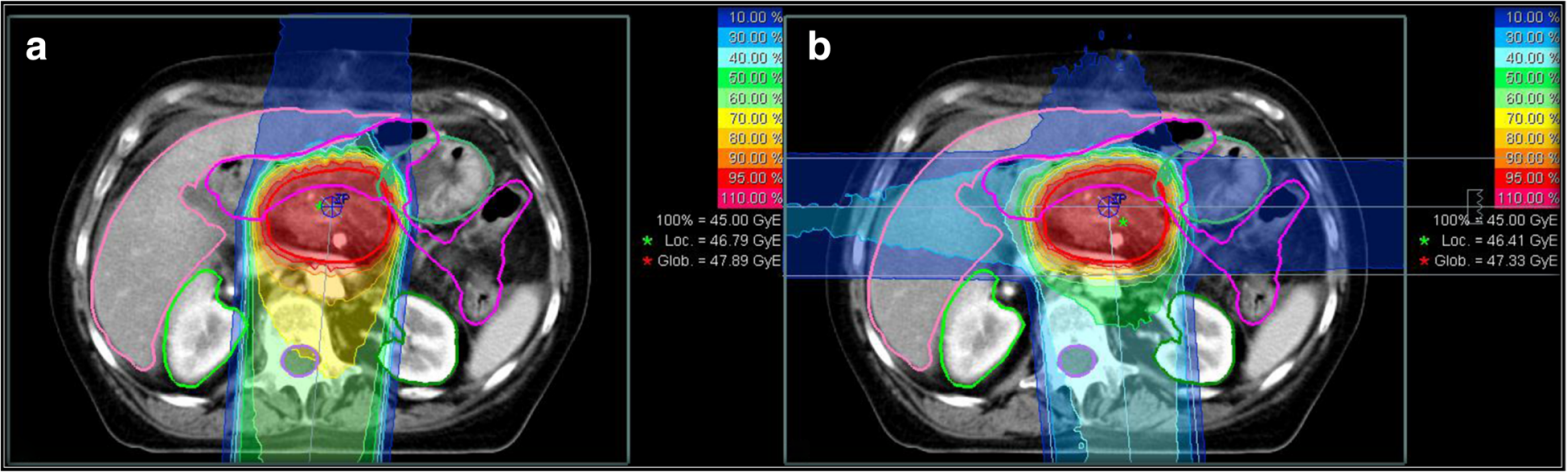
Two relevant Field-setups of carbon ion beams of pancreatic cancer: **a** One-Field Setup with a single posterior field. **b** Three-Fields Setup with posterior, right lateral and left lateral fields (decreasing weighting factors of Single Beam Optimization)

The central position of pancreatic cancer is a major problem of radiotherapy in general. With ion beam therapy being highly conformal with sharp dose gradients, and at the same time being very time consuming, the number of beams is restricted to a realistic level, in order to preserve the advantages over photon radiotherapy. Three to four fields ion beam radiotherapy have already been used in Japanese trials [27, 38]. But, Shiomi et al. could show an advantage of three-fields setups, although one has to be clear about the fact, that both setups use beams in anatomic regions with high intra- and interfractional dosimetric uncertainties (e.g. colon). Other possible, realistic field setups are two-fields setups from posterior and one single (posterior) field setup. In this case, dose exposure to radiosensitive organs such as the spinal cord has to be critically analyzed. In-silico studies at HIT showed the superiority of three-fields setups (Fig. 1) [39]. The one-field setup with a single posterior field was also promising, although the maximum doses in the myelon were thoroughly high [40]. This can be due to many reasons, however, one explanation can be overdosage in the Bragg Peak region potentially due to higher biological effects in the distal edge of the Bragg Peak of particle beams.

### Plan optimization

Generally, the different TPS offer two kinds of planning: in case of the TPS “*syngo RT Planning*” (Siemens, Erlangen, Germany) they are called *single field uniform dose optimization* (SBO, Single Beam Optimization) or *multiple field optimization* (IMPT, Intensity Modulated Particle Therapy). Both tools are based on intensity modulation, but SBO includes relative weighting factors for each beam. Each beam is optimized independently to a homogenous dose level and all beams add up to 100% of the prescribed dose. IMPT directly integrates all beams and optimizes simultaneously. So, IMPT is able to compensate bad characteristics of one field with another, nevertheless being at the same time prone to distance uncertainty of the ion beam [41–43].

### Biological plan optimization

In case of proton radiotherapy a general RBE of 1.1 is assumed but still remains questioned, at least in SOBP region [34, 44].

So far, treatment planning and application of carbon ion beams is usually based on a fixed RBE or $$ \raisebox{1ex}{$\alpha $}\!\left/ \!\raisebox{-1ex}{$\beta $}\right. $$-value, although carbon ion radiotherapy is highly dependent on the biological characteristics of the tissues (represented by the $$ \raisebox{1ex}{$\alpha $}\!\left/ \!\raisebox{-1ex}{$\beta $}\right. $$-value). At HIMAC an average RBE of 3.0 is usually assumed at mid-SOBP, at HIT clinical practice usually assumes a general $$ \raisebox{1ex}{$\alpha $}\!\left/ \!\raisebox{-1ex}{$\beta $}\right. $$-value of 2 Gy - this $$ \raisebox{1ex}{$\alpha $}\!\left/ \!\raisebox{-1ex}{$\beta $}\right. $$-value has been chosen, in order to represent a worst-case calculation of the risk of high grade myelopathy [45–47]. RBE values in the established hypofractionated dose prescription setting are about 3 in the target volume and vary from about 2–7 in the OARs, depending on the dose distribution.

**Fig. 2:**
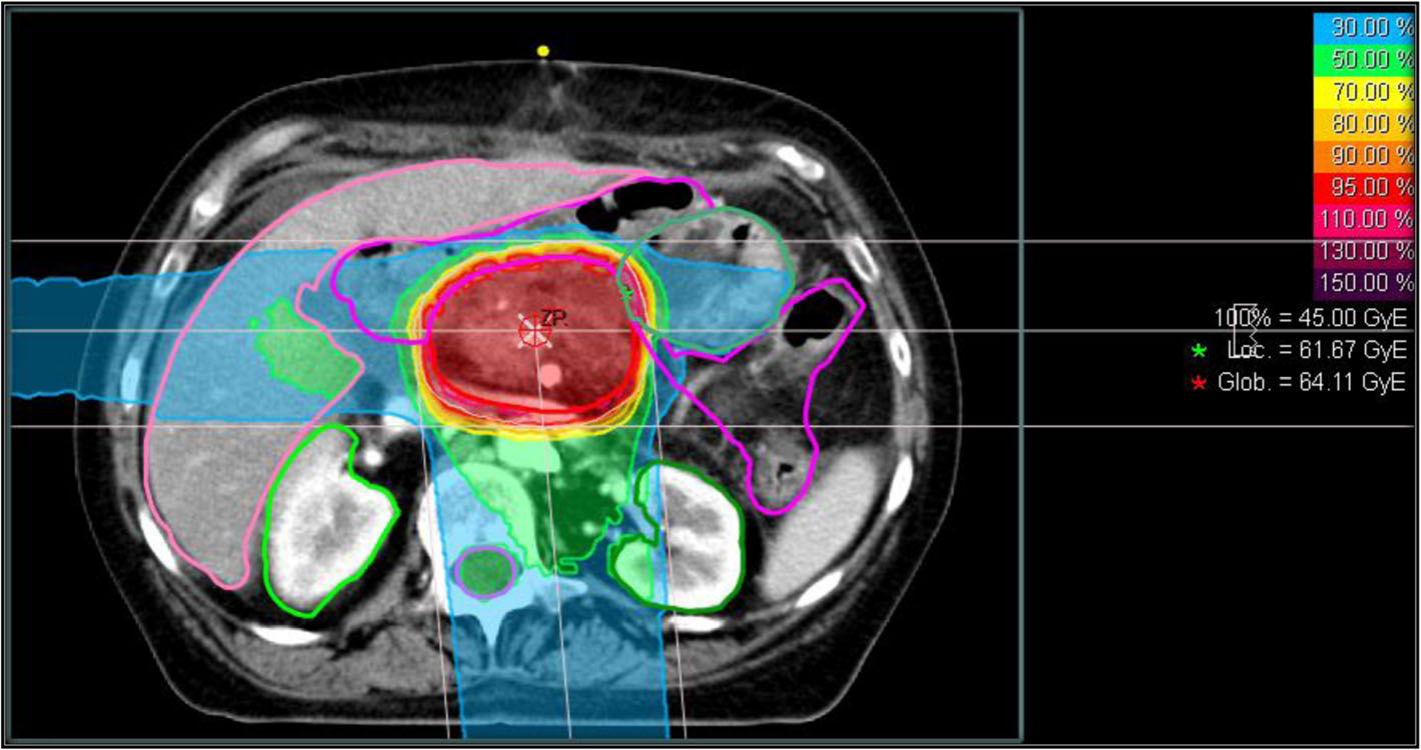
Three-Fields setup of carbon ion beams of pancreatic cancer: dose distribution after allocating tissue specific α/β-values

However, in order to increase the accuracy of treatment planning, one has to take all the tissues’ specific $$ \raisebox{1ex}{$\alpha $}\!\left/ \!\raisebox{-1ex}{$\beta $}\right. $$-values into account, and LEM at HIT is able to do so. An in-silico study could show its establishment and the tissue specific dose distribution in case of LAPC (Fig. 2) [48]. The specific $$ \raisebox{1ex}{$\alpha $}\!\left/ \!\raisebox{-1ex}{$\beta $}\right. $$-value for pancreatic cancer of 4.5 Gy has been identified by El-Shafie et al. at HIT [14].

In summary, the integration of tissue specific $$ \raisebox{1ex}{$\alpha $}\!\left/ \!\raisebox{-1ex}{$\beta $}\right. $$-values increases the accuracy of plan optimization. Nevertheless, tolerance doses of the normal tissues are not well defined for ion beam irradiation - so far, the $$ \raisebox{1ex}{$\alpha $}\!\left/ \!\raisebox{-1ex}{$\beta $}\right. $$-values are extrapolated almost exclusively from photon-based data. Further research in the exact radiobiological characteristics after ion beam radiotherapy are needed, in order to implement tissue specific biological treatment planning in daily clinical routine.

## Treatment challenges

### Organs at risk

Surely, treatment concepts are highly influencing the clinical outcome, but target volumes, field setups and the consecutive dose distributions can directly be translated into different risk profiles. Posterior fields deposit high doses to the spinal cord and the kidneys, right lateral fields result in dose exposure to the liver. Higher doses in intestinal structures are generated by left lateral and anterior fields.

A possible single posterior field setup is of major concern, because of its steep RBE-increase at the distal end of the SOBP, leading to unexpected high doses to the small intestine. Based on SBRT trials, less than 4%/ 5 ccm of the stomach should receive more than 22.5 Gy [49]. With ion beam therapy being accompanied by RBE-increase at the distal end, this constraint might be exceeded. There is a retrospective analysis of a small cohort with promising results after high dose proton radiotherapy with little adverse side effects [50]. Unfortunately, M.D. Anderson Cancer Center and Takatori et al. reported several events of intestinal ulcerations after high dose proton radiotherapy of pancreatic cancer [24, 51–54]. So, the stomach/small bowel is one of the main OARs in ion beam therapy of pancreatic cancer – there are up to 50% radiation-induced ulcers after high dose proton radiotherapy with concurrent gemcitabine application [24]. Similarly, Terashima et al. reported high intestinal toxicity after aggressive simultaneous radiochemotherapy [55]. Shinoto et al. could show, that a possible constraint for ulcerations of the upper gastrointestinal tract might be D2ccm < 46 Gy(RBE) [23].

High dose deposition in the colon might also result in clinically relevant complications, which is why Terashima et al. divided their patient collective into those with contact to the intestines and those without, thus applying 50 Gy(RBE) or 70.2 Gy(RBE) to the target volume [55]. Another possibility might be simultaneous integrated protection in the target volume, which has also been used by Terashima et al. [55]. With regard to gastrointestinal complications, at HIT there is experience on comparable dose protocols with intestinal structures adjacent to the target volume, such as carbon ion therapy of sacral chordoma and locally recurrent rectal cancer, where no higher gastrointestinal toxicities were recorded [56, 57].

### Intra- and interfractional variability and dosimetric changes

As described before, ion beam radiotherapy is on the one hand characterized by very sharp dose gradients, but on the other hand these sharp dose gradients lead to great challenges in case of dosimetric uncertainties. Robustness in ion beam therapy of pancreatic cancer is dependent on patient immobilization, target volume, beam optimization, beam setups, interfractional and intrafractional changes:

Due to tumor and OAR movements during radiotherapy a robust patient immobilization setup has to be established, especially in highly precise hypofractionated particle therapy [58, 59]. To date, no general recommendation on the most reliable setup in pancreatic cancer patients can be given, but the different setups lead to significant movement reductions of the tumor, the pancreas in total and the OARs compared to without any immobilization [60–62]. Further studies on the exact tumor movement by the use of 4D–MRI (magnetic resonance imaging) and 4D–CT scans have to be conducted, in order to improve treatment planning and enable dose escalation in particle therapy.

In case of photon radiotherapy these challenges resulted in the PTV concept and obviously, this has to be taken over in ion beam therapy, despite of limiting the advantages of the sharp dose gradients with regard to dose exposure to the OARs [37]. Nevertheless, the exact margins of the different treatment volumes have to be re-evaluated for ion beam radiotherapy.

Based on the central position in the abdomen, pancreatic cancer is totally surrounded by OARs, and that’s the reason why ion beam therapy of abdominal organs, and especially pancreatic cancer is very complex. Inter- and intraindividual (inter- and intrafractional) changes in organ motion and intestinal fillings anterior and left laterally of the target volume are a great challenge for robust ion beam therapy. Kumagai et al. reported an analysis of passive scattered carbon ion beams, showing that anterior-posterior and left-right field setups cause the highest dose affections [63]. Therefore, the established 4- and 3-fields setups have to be critically analyzed. Steitz et al. at HIT could also show that SBO plan optimization is able to compensate interfractional bowel movement in case of dose deposition in the target volume [64].

Intrafractional movements due to breathing lead to a decrease in robustness, possibly resulting in overdosage in OARs and underdosage in the target volume [65–67]. As breathing itself obviously influences all organs and tissues, gating might be a solution. Taniguchi et al. analyzed doses in duodenum and stomach in patients with LAPC treated with a five-fraction protocol: results show a decreasing dose exposure of the OARs during expiration compared to inspiration [67]. Furthermore, Fontana et al. could show, that the expiration phase also has the highest stability of pancreatic cancer motion in 4D–MRI [60]. So, including breathing phases in treatment planning and gating in general is highly promising in pancreatic cancer patients [68].

With regard to the above-mentioned robustness challenge, one could assume that ion beam therapy of pancreatic cancer should be conducted by the use of a single posterior field. A single posterior beam might be robust, but small rotations of the processi transversi can lead to different dose depositions in the pancreatic cancer. Nevertheless, Batista et al. have presented data about pancreatic cancer, that supported this hypothesis. A single posterior field and two oblique posterior fields are superior in case of robustness [40]. But, dose deposition by a single field leads to high integral dose in its trajectory, resulting in high dose deposition in the spinal cord itself, probably violating general QUANTEC (Quantitative Analyses of Normal Tissue Effects in the Clinic) constraints [69].

However, intra- and interfractional changes are not totally understood. We need re-planning scenarios, as slight changes result in significant dose variations especially in case of scanned particle therapy, which is used at HIT [63, 67, 70, 71]. Of course, there are advantages of scanning, e.g. in case of conformal and highly precise dose deposition in the target volume [30]. But active scanning is at the same time highly vulnerable due to robustness problems, such as interplay effects. At least, Richter et al. at HIT were able to show, that fractionation is a potential tool to reduce dose inhomogeneity by interplay effects [66, 72]. This in return promotes normofractionated radiotherapy, instead of the established hypofractionated dose regimes. Additionally other methods of compensation, such as tracking, are currently under critical investigation and might provide additional benefit for moving targets.

## Dose delivery and clinical outcome

The facilities around the world generally accelerate the ion beams in different ways. In case of pancreatic cancer, the HIT Linac-Synchrotron needs to assemble ion energies of 160 MeV for proton beams and up to 430 MeV/u for carbon ion beams [73].

Ion beam has the great potential to increase secondary resectability and prognosis of LAPC patients, but at the same time it has to be critically evaluated with regard to adverse side effects. Despite of the risk of radiation induced complications, proton radiotherapy of 50 Gy(RBE) with concurrent gemcitabine and proton radiotherapy of 50.4–59.4 Gy(RBE) with concurrent capecitabine are well tolerated [55, 74]. Nevertheless, there are also reports on combined treatment regimes with proton beam therapy and concurrent gemcitabine, that show radiation-induced ulcers in stomach and duodenum in approximately 50% of all treated patients [24]. Furthermore, preoperative short-course chemoradiotherapy with proton beams (25 Gy(RBE) in 5 fractions) and capecitabine could prove its feasibility in resectable pancreatic cancer [75].

Carbon ion beams in particular offer a higher RBE compared to proton beams, which might further increase the response rate and resectability of LAPC, and decrease radiation induced complications [14–16]. There are encouraging clinical results of hypofractionated carbon ion radiotherapy of up to 55.2 Gy(RBE) and concurrent gemcitabine of LAPC [38, 76, 77]. A phase I trial of neoadjuvant carbon ion radiotherapy of up to 36.8 Gy(RBE) in patients with resectable pancreatic cancer resulted in 5-year overall survival rates of 42 and 52% for all patients and those with surgery afterwards [38]. In general, it remains unclear, whether high dose or high RBE are more beneficial in case of pancreatic cancer. Nevertheless, we are in desperate need of the latest advances in radiation oncology to improve the prognosis of pancreatic cancer. Modern radiotherapy techniques such as SBRT with high fraction doses and carbon ion beams with high RBE values are promising – but, to date there is no evidence of improved prognosis by the use of SBRT or ion beam therapy, even in the setting of combined chemoradiotherapy. Randomized trials about modern photon radiotherapy and ion beam therapy with and without simultaneous chemotherapy are needed.

## Summary

Ion beam therapy of pancreatic cancer is very complex. There are a lot of challenges to overcome. First clinical results are very promising, as presumed before, with regard to preclinical analysis of particle beams and pancreatic cancer. The KFO “Schwerionentherapie” at HIT was able to do the first steps in this research topic. Nevertheless, robust treatment planning and dose delivery has to be ensured and the optimal treatment concepts - also whether or not particle therapy should be combined with systemic agents- are still to be identified in future projects.

## Abbreviations

CT: computed tomography
CTV: clinical tumor volume
GSI: Gesellschaft für Schwerionenforschung
GTV: gross tumor volume
HIT: Heidelberg Ion Beam Therapy Center
IMPT: Intensity Modulated Particle Therapy
IMRT: intensity-modulated radiotherapy
LAPC: locally advanced pancreatic cancer
LEM: Local Effect Model
LET: linear energy transfer
OAR: organs at risks
QUANTEC: Quantitative Analyses of Normal Tissue Effects in the Clinic
RBE: relative biological effectiveness
SBO: Single Beam Optimization
SBRT: stereotactic body radiotherapy
TPS: treatment planning systems
VMAT: volumetric-modulated arc therapy
